# Cellulite as a Possible Biomarker of Metabolic Dysfunction: Redefining Body Remodeling in the Glucagon-Like Peptide-1 Era Through the Cellulite Removal Medical Protocol-G21 Concept

**DOI:** 10.7759/cureus.110805

**Published:** 2026-06-13

**Authors:** Nikos Adamidis, Sofia Adamidi, Vasiliki E Georgakopoulou, Nektaria Papadopoulou, Sotirios Adamidis

**Affiliations:** 1 First Department of Internal Medicine, Sismanogleio Hospital, Athens, GRC; 2 Internal Medicine, Charlton Memorial Hospital, Massachusetts, USA; 3 Pathophysiology/Pulmonology, Laiko General Hospital, Athens, GRC; 4 Endocrinology, National and Kapodistrian University of Athens, Athens, GRC; 5 First Department of Internal Medicine, Athens Medical Group, Athens, GRC

**Keywords:** cellulite, crmp-g21, metabolic-dermal axis, metabolic dysfunction, tirzepatide

## Abstract

Cellulite has traditionally been regarded as a localized aesthetic condition; however, it may also reflect interactions between adipose tissue biology, dermal architecture, microcirculation, extracellular matrix remodeling, inflammation, and metabolic status. This editorial proposes the metabolic-dermal axis as a conceptual framework for understanding cellulite beyond its surface appearance. In this context, insulin resistance, chronic low-grade inflammation, adipose tissue dysfunction, impaired microvascular function, and altered collagen organization may contribute to the development or persistence of cellulite-like changes. The glucagon-like peptide-1 receptor agonist era, including therapies such as tirzepatide, has transformed obesity and metabolic medicine by improving weight, adiposity, insulin sensitivity, and cardiometabolic risk. Nevertheless, weight loss and metabolic improvement do not necessarily restore dermal structure, skin firmness, or tissue quality. Persistent cellulite and skin laxity after substantial weight reduction highlight the need for approaches that address both systemic metabolic health and local dermal remodeling. The Cellulite Removal Medical Protocol-G21 (CRMP-G21) is presented here as a proposed integrative concept rather than a validated therapeutic standard. It aims to combine metabolic optimization with targeted dermal restoration strategies. Further clinical studies are required to determine whether cellulite can serve as a meaningful clinical marker of metabolic-dermal dysfunction and whether combined systemic and local interventions improve patient outcomes.

## Editorial

Cellulite has traditionally been interpreted as a cosmetic imperfection, a superficial alteration of skin texture mainly relevant to aesthetic medicine. However, this limited interpretation no longer reflects the biological complexity of the condition. Cellulite is now recognized as a multifactorial condition involving structural, vascular, inflammatory, and metabolic components. Independent literature has linked cellulite to alterations in subcutaneous tissue architecture, adipose tissue protrusion into the dermis, connective tissue remodeling, edema, fibrosis, impaired microcirculation, and extracellular matrix changes [1]. These mechanisms support the view that cellulite cannot be fully explained as a simple cosmetic surface irregularity, although its role as a marker of systemic metabolic dysfunction remains hypothesis-generating and requires further validation.

Increasing evidence suggests that cellulite may reflect deeper structural and metabolic disturbances involving subcutaneous adipose tissue, dermal architecture, connective tissue remodeling, microcirculatory impairment, inflammation, oxidative stress, and insulin resistance-related adipose dysfunction [1]. In this sense, cellulite should not be viewed merely as an aesthetic concern, but as a potential clinical correlate expressed through the skin.

This conceptual shift is clinically important. If cellulite is understood only as a local skin irregularity, treatment remains restricted to surface-directed approaches. If, however, cellulite is interpreted as a metabolic-dermal interface, the therapeutic target changes. The skin may be considered a visible clinical site where adipose tissue biology, extracellular matrix integrity, microvascular function, dermal structure, and systemic metabolic health interact. This broader biological interpretation remains conceptual, but it is supported by evidence showing that cellulite is associated with alterations in connective tissue structure, microcirculation, dermal-subcutaneous interactions, and extracellular matrix organization [1].

This is the philosophy behind the Cellulite Removal Medical Protocol-G21 (CRMP-G21): we do not treat cellulite; we treat the biology behind it. CRMP-G21 is proposed as a conceptual and integrative framework in which cellulite management is not reduced to fat reduction, skin tightening, or temporary smoothing of the skin surface. Instead, it is reframed as a potential biological remodeling strategy. The proposed framework combines systemic metabolic correction with local dermal restoration, aiming to address both internal metabolic drivers and the external tissue expression of cellulite. Importantly, CRMP-G21 should not be interpreted as an established evidence-based standard of care, but as a hypothesis-generating model that requires independent validation.

This approach is particularly relevant in the glucagon-like peptide-1 (GLP-1) era. Incretin-based therapies, and especially tirzepatide, have transformed the medical management of obesity and metabolic dysfunction. Tirzepatide, a dual glucose-dependent insulinotropic polypeptide (GIP) and GLP-1 receptor agonist, has demonstrated substantial effects on body weight, waist circumference, adiposity, insulin resistance, glycemic control, and broader cardiometabolic risk in randomized clinical trials and real-world observational studies [2-5].

The efficacy of tirzepatide for obesity management has been established in large randomized controlled trials. In SURMOUNT-1, tirzepatide produced substantial and clinically meaningful weight reduction in adults with obesity or overweight without diabetes, supporting its role as a major pharmacologic therapy for obesity [4]. In SURMOUNT-4, continued tirzepatide treatment maintained and further enhanced weight reduction, whereas treatment withdrawal was associated with significant weight regain [5]. These independent trial data provide the broader evidence base for tirzepatide as a metabolic therapy, while real-world studies offer complementary observational information regarding body composition, metabolic parameters, and clinical outcomes [2,3].

In a short-term real-world prospective study, tirzepatide was associated with rapid reductions in body weight, waist circumference, and fat mass within 30 days, while fat-free mass and total body water were relatively preserved [2]. Longer-term real-world evidence further supports tirzepatide as a comprehensive metabolic therapy, with one-year improvements in adiposity, glycemic control, insulin resistance, inflammatory markers, liver enzymes, and sleep-related outcomes, again with relative preservation of lean mass [3]. These observational findings should be interpreted as supportive real-world evidence rather than definitive proof of efficacy across all body remodeling outcomes.

However, metabolic success creates a new clinical challenge. In the GLP-1 era, many patients can achieve substantial weight loss and meaningful metabolic improvement. Yet weight loss alone does not necessarily restore dermal architecture, extracellular matrix organization, microcirculatory function, or skin quality. A patient may lose fat mass, reduce waist circumference, improve insulin resistance, and lower inflammatory burden, while still presenting with skin laxity, tissue irregularity, cellulite persistence, or altered dermal texture. This creates the modern paradox of metabolic success and dermal challenge.

Therefore, post-GLP-1 body remodeling must move beyond the scale. The endpoint should not be weight loss alone, but the restoration of tissue quality. Body remodeling in this new era may require a dual objective: systemic metabolic correction and local dermal restoration. Metabolic therapy improves the internal biological environment, while tissue-targeted interventions may be needed to address visible structural consequences that persist at the skin surface.

The metabolic-dermal axis provides a useful conceptual framework for this new approach. While tirzepatide can improve systemic metabolic dysfunction, obesity trials do not directly demonstrate improvement in cellulite or dermal quality [4,5]. Therefore, extrapolation from weight-loss outcomes to cellulite outcomes should remain cautious. Cellulite involves not only adiposity, but also fibrous septae, dermal-subcutaneous interactions, collagen organization, oxidative stress, microvascular dysfunction, lymphatic stasis, and extracellular matrix remodeling [1]. For this reason, local dermal restoration may remain relevant even when systemic metabolic improvement has been achieved.

The G21 component of the CRMP-G21 concept is proposed as a multi-pathway topical approach targeting local biological mechanisms involved in cellulite pathophysiology. Its rationale includes support of microcirculation, modulation of inflammation and oxidative stress, support of collagen synthesis, and preservation of extracellular matrix integrity. However, the specific clinical contribution of G21 requires further independent evaluation. At present, it should be regarded as part of a proposed investigational framework rather than as a validated therapeutic standard.

When combined with tirzepatide, CRMP-G21 is proposed to create a dual-axis strategy. Tirzepatide addresses systemic metabolic dysfunction, including excess adiposity, insulin resistance, inflammation, and cardiometabolic risk [2-5]. G21 is intended to address local dermal and subcutaneous tissue alterations that remain visible as cellulite. The combination, therefore, represents a proposed model of body remodeling: metabolic correction from within and dermal restoration from the surface. However, this model remains investigational and should not be considered validated until tested in independent prospective studies.

In this context, the future of body remodeling after GLP-1-based weight loss should not be defined only by kilograms lost. It should also consider the quality of biological and structural restoration achieved. A patient who loses weight but retains cellulite, dermal laxity, or poor tissue quality may have achieved metabolic improvement but incomplete tissue remodeling. Conversely, a patient who receives only local cellulite treatment without addressing insulin resistance, adipose dysfunction, or systemic inflammation may experience superficial improvement without correction of the underlying biology.

The CRMP-G21 concept attempts to bridge this gap. It proposes that cellulite should be approached not as an isolated cosmetic defect, but as a possible visible expression of the metabolic-dermal axis. The skin is not separate from metabolic disease; it may be one of the tissues through which metabolic disturbances become clinically visible. Cellulite, therefore, may function as a visible correlate of systemic and local dysfunction, reflecting interactions between metabolism, adipose tissue, microcirculation, connective tissue, and dermal integrity [1]. This interpretation, however, remains theoretical and requires validation.

Redefining body remodeling in the GLP-1 era requires a new clinical language. The goal is no longer simply to reduce fat, flatten the skin, or improve appearance. The broader aim is to restore biological function, preserve lean mass, improve adipose tissue behavior, support dermal architecture, enhance microcirculation, and recover tissue quality. In this framework, cellulite is not necessarily the primary target, but rather a visible signal that may reflect a deeper biological process.

The central message of CRMP-G21 is therefore that cellulite management may need to address the biology behind the visible skin changes. This philosophy repositions cellulite from a purely cosmetic inconvenience to a potentially meaningful expression of the metabolic-dermal axis. In the GLP-1 era, future approaches to body remodeling may benefit from integrating metabolic improvement with dermal restoration, but this concept requires rigorous scientific validation before it can be adopted as a standard clinical approach.

Despite the biological plausibility of the metabolic-dermal axis, several limitations must be acknowledged. This editorial presents a conceptual framework and should not be interpreted as definitive clinical evidence. Cellulite severity and treatment response are influenced by multiple confounding factors, including age, sex, baseline body mass index, adiposity distribution, hormonal status, menopausal status, pregnancy history, genetic predisposition, lifestyle habits, physical activity, diet, smoking, skin thickness, connective tissue characteristics, and baseline cellulite grade. These variables may independently affect dermal architecture, microcirculation, adipose tissue behavior, extracellular matrix remodeling, and visible cellulite outcomes. Therefore, although CRMP-G21 proposes an integrated approach combining systemic metabolic correction with local dermal restoration, its clinical value requires confirmation in prospective controlled studies with adequate sample size, standardized cellulite assessment tools, defined patient populations, and appropriate adjustment for relevant confounders. Future research should also distinguish between the effects of weight loss, metabolic improvement, topical or local interventions, and their combination on cellulite severity, skin quality, dermal laxity, and patient-reported outcomes.

A further limitation is that part of the rationale for CRMP-G21 is based on preliminary work conducted by the authors’ group, which may introduce potential author-related bias. Therefore, the proposed framework requires confirmation through independent, peer-reviewed, prospective controlled studies before it can be considered clinically established.
